# Spontaneous epiglottic abscess: pathophysiology and airway management options

**DOI:** 10.1093/jscr/rjad481

**Published:** 2023-08-22

**Authors:** Fahad Z Alotaibi

**Affiliations:** Department of Otolaryngology—Head and Neck Surgery, Imam Mohammad Ibn Saud Islamic University IMSIU, Riyadh 13317-4233, Saudi Arabia

**Keywords:** abscess, stridor, diabetes, epiglottitis

## Abstract

Laryngeal abscesses are rare in the modern antibiotic era. This report presents a novel case of an epiglottic abscess in a patient with diabetes who developed respiratory distress and was managed by awake intubation in the emergency room followed by transoral incision and drainage of the abscess and tracheostomy. Full recovery after 1 week of intravenous antibiotic treatment was observed. Surgical intervention is necessary for treatment and culture-directed antimicrobial therapy. Poorly controlled diabetes is a newly described risk factor for spontaneous epiglottic abscess development.

## INTRODUCTION

Acute laryngeal infections with abscess formation are traditionally divided into primary and secondary infections. The former usually starts after precipitating events such as trauma or localized bacterial infection including Hemophilus influenza type B (HiB)-related epiglottitis, whereas the latter occurs when an infection from tonsillitis or nasopharyngitis spreads to the larynx [1]. An epiglottic abscess is a rare and infrequent sequela of epiglottitis, occurring in only 4% of epiglottitis cases [2]. Since the introduction of the HiB vaccine, the prevalence rate of both epiglottitis and epiglottic abscess has dramatically decreased. An epiglottic abscess requires immediate diagnosis and treatment because it is associated with a high mortality rate (30%) due to abscess rupture, hemorrhage and airway obstruction [2].

Airway obstruction is the most feared complication of epiglottitis, whereas descending necrotizing mediastinitis and epiglottic abscesses are unusual complications that may necessitate surgical intervention [3]. Laboratory investigations and radiological modalities, such as lateral soft tissue neck radiography and computed x (CT) scan, can facilitate confirmation of the diagnosis of an epiglottic abscess [4]. Treatment involves the urgent establishment of an artificial airway along with abscess drainage and subsequent intravenous antibiotic treatment [5]. We present a rare case of a patient with diabetes with a spontaneous epiglottic abscess, with a detailed description and discussion of airway management in this type of situation.

## CASE PRESENTATION

**Figure 1:**
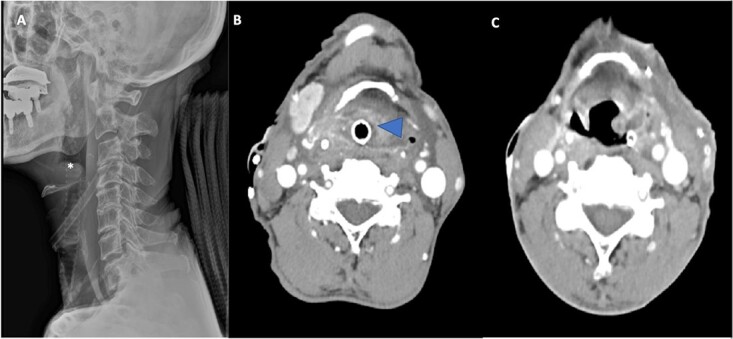
(**A**) Lateral radiograph of the neck shows a swollen epiglottis (^*^). (**B**) Computed tomography axial cut shows a collection in the paraglottic space (arrowhead) and parapharyngeal space extenuation with effaced pharyngeal and supraglottic lumina due to epiglottic enlargement. (**C**) Computed tomography axial cut at Day 7 post-operatively reveals a marked reduction in the size of the collection.

**Figure 2:**
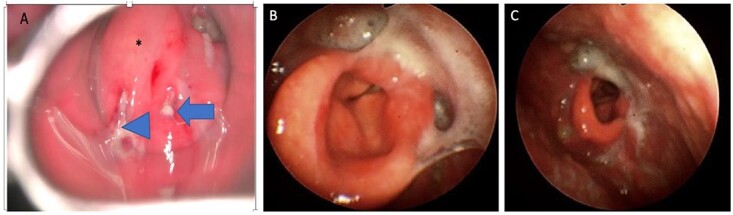
(**A**) Direct laryngoscopic examination shows a swollen epiglottis (^*^) with pus extruding at the level of the right and left aryepiglottic folds as indicated by arrowhead and arrow, respectively. (**B**) Transnasal laryngoscopic examination of the larynx at Day 7 post-operatively revealed a normal-shaped epiglottis (^*^) with a visible glottic airway during adduction and (**C**) abduction.

A 72-year-old man with diabetes presented to the emergency department with dyspnea, stridor and odynophagia that suddenly started 3 h earlier. Clinical examination revealed that the patient was febrile with hypoxia, hoarseness and drooling. Flexible fiber optic rhinopharyngo-laryngoscopy showed pooling of secretions in the pyriform sinuses, a swollen and edematous epiglottis, poor visualization of the glottis and reduced vocal cord movement. A lateral neck radiograph revealed a swollen epiglottis and, due to his respiratory distress, the patient was intubated by the emergency physician in the emergency room; CT of the neck with intravenous contrast revealed a multiloculated abscess involving the epiglottis, and paraglottic as well as parapharyngeal spaces (Fig. 1A and B). Laboratory workup revealed an elevated white blood cell count and inflammatory markers. The patient was transferred to the operating room for microlaryngoscopic examination, combined transoral and transcervical incision, neck abscess drainage and tracheostomy. A Klinssaer laryngoscope was used to expose the supraglottic and glottic regions. A gush of pus was observed during laryngoscope insertion into the vallecula and epiglottis, and two small incisions were made at the level of the ventricular folds using a laryngeal knife (Fig. 2A). A swab was collected and sent for bacteriological and mycological examination. The tissue biopsies were performed using cup forceps. Subsequently, a transcervical approach to drain the left parapharyngeal space was begun, with an incision made two fingerbreadths below the left mandible, after which the subplatysmal flaps were elevated superiorly and inferiorly. The parapharyngeal space was accessed after medial retraction of the submandibular gland and dissection above the posterior belly of the digastric muscle, where pus collection was drained. Warm saline irrigation in addition to diathermy was used to achieve hemostasis, and a negative pressure drain system was inserted prior to multilayer wound closure. Finally, a tracheostomy was performed utilizing a 2-cm anterior neck incision at the level of the third tracheal ring. At the end of the procedure, a nasogastric tube was inserted to ensure enteral feeding during the recovery period. After an infectious disease consultation, the patient was started on ceftriaxone and metronidazole for 1 week. Marked improvement in the patient’s symptoms was observed. The flexible scope showed improved visualization and mobility of the supraglottic and glottic areas (Fig. 2B and C), and decannulation was performed on day 4 after passing a trial. A CT scan repeated 1 week post-operatively revealed marked resolution of the abscess (Fig. 1C). The patient was administered oral antibiotics for another week. Clinical follow-up after 1 month was unremarkable.

## DISCUSSION

Epiglottic abscess is a rare disease in the modern era, owing to the availability of a wide range of antibiotics and modern investigation techniques [6]. It represents a serious complication of epiglottitis and exclusively affects adults [7, 8]. Patient populations at risk of epiglottic abscess include immunocompromised individuals [9]. Mortality due to epiglottic abscesses is three times more likely in males than females [10]. In the present study, we report the case of an epiglottic abscess in an older man with type 2 diabetes mellitus (DM), and both advanced age and DM are associated with an immunocompromised state. Patients with diabetes are at an increased risk of developing epiglottic abscesses due to a lower immune system and poor perfusion, which impair the healing process. DM was also identified by Berger *et al*. and Ridgeway *et al*. [11] as a risk factor for the development of epiglottic abscesses. Similarly, the patient described in our case report is a known case of type 2 DM that developed an epiglottic abscess. Although *Streptococcus* and *Staphylococcus* species are the most commonly identified causes of adult epiglottic abscesses [12], patients with diabetes may be affected by rare pathogens such as *Neisseria meningitidis* and *Aeromonas hydrophila* [13]. In the present case, swab and tissue cultures showed a mixed bacterial growth with Gram-positive rods.

Fever, sore throat, odynophagia, dysphagia, neck discomfort, stridor, drooling and shortness of breath are common signs and symptoms of acute infections, as observed in our case. Patients are at risk of rapid airway compromise. Therefore, monitoring patients for signs of oxygen desaturation, worsening stridor, adoption of a tripod posture and drooling is crucial [14].

Different investigative modalities can be used if a patient does not experience severe respiratory distress. Flexible fiberoptic laryngoscopy is a quick, safe and accurate method to quickly assess the supraglottic and glottic areas as well as airway patency. A lateral neck soft tissue radiograph showed an enlarged epiglottis (thumb sign), which can also be observed in acute epiglottitis. CT scan of the neck with intravenous contrast is helpful in evaluating the airway and differentiating acute epiglottitis from epiglottic abscess; accordingly, it represents the best modality for accurate diagnosis and follow-up of the condition [15]. Similarly, in our patient, multiloculated ring-enhancing lesions and fluid in the paraglottic, glottic and parapharyngeal areas were observed on a CT scan of the neck with intravenous contrast administration.

This case highlights the importance of a multidisciplinary approach in patient care as well as the use of various diagnostic and therapeutic techniques to manage epiglottic abscesses. Airway control was performed in an operating room. Awake intubation using a video laryngoscope, bronchoscopy-guided intubation or tracheostomy under local anesthesia are all possible airway management strategies [10]. After securing the airway, the definitive treatment of epiglottic abscess is incision and drainage [3, 16]. This is in contrast to the management of acute epiglottitis, in which the management is only supportive and comprises the administration of humidified oxygen, nebulized adrenaline and empiric antibiotic therapy to cover suspected causative organisms [17]. Antimicrobial treatment usually resolves edema within 2–3 days.

Antibiotics with wide coverage should be selected; a combination of third-generation cephalosporins and metronidazole provides coverage against Gram-positive, Gram-negative and anaerobic bacteria [18]. Steroid use has been shown to result in shorter hospital stays [19]. Additional treatments include oxygen, intravenous fluid and racemic epinephrine. Racemic epinephrine has been proven useful in treating epiglottic abscesses as it helps in reducing associated airway edema. However, it should be used with caution in older patients with myocardial ischemia or arrhythmia [19].

In conclusion, acute laryngeal abscesses are considered uncommon in the current era due to vaccination efforts, novel antibiotics and early diagnosis of epiglottitis. This case serves as a crucial reminder for physicians to check for epiglottitis in patients presenting with these symptoms to prevent the progression of epiglottitis into an epiglottic abscess. Early diagnosis and treatment of these manifestations can reduce the risk of airway loss, which can be fatal. Regular monitoring of blood glucose levels and good diabetes control can help reduce the risk of epiglottic abscess development and accelerate recovery.

## Data Availability

All data used in this study are available from the corresponding author upon request.
